# Investigating Biomarkers for *USH2A* Retinopathy Using Multimodal Retinal Imaging

**DOI:** 10.3390/ijms23084198

**Published:** 2022-04-11

**Authors:** Jasdeep S. Gill, Vasileios Theofylaktopoulos, Andreas Mitsios, Sarah Houston, Ahmed M. Hagag, Adam M. Dubis, Mariya Moosajee

**Affiliations:** 1 Institute of Ophthalmology, University College London, London EC1V 9EL, UK; j.gill.17@ucl.ac.uk (J.S.G.); v.theofylaktopoulos@ucl.ac.uk (V.T.); andreas.mitsios@ucl.ac.uk (A.M.); sarah.houston.16@ucl.ac.uk (S.H.); a.hagag@ucl.ac.uk (A.M.H.); a.dubis@ucl.ac.uk (A.M.D.); 2 NIHR Moorfields Biomedical Research Centre, Moorfields Eye Hospital NHS Foundation Trust, London EC1V 2PD, UK; 3 Global Business School for Health, University College London, London WC1E 6BT, UK; 4 Great Ormond Street Hospital for Children NHS Foundation Trust, London WC1N 3JH, UK

**Keywords:** *USH2A*, retinitis pigmentosa, Usher syndrome, fundus autofluorescence, spectral-domain optical coherence tomography, adaptive optics scanning laser ophthalmoscopy

## Abstract

Pathogenic mutations in *USH2A* are a leading cause of visual loss secondary to non-syndromic or Usher syndrome-associated retinitis pigmentosa (RP). With an increasing number of RP-targeted clinical trials in progress, we sought to evaluate the photoreceptor topography underlying patterns of loss observed on clinical retinal imaging to guide surrogate endpoint selection in *USH2A* retinopathy. In this prospective cross-sectional study, twenty-five patients with molecularly confirmed *USH2A*-RP underwent fundus autofluorescence (FAF), spectral-domain optical coherence tomography (SD-OCT) and adaptive optics scanning laser ophthalmoscopy (AOSLO) retinal imaging. Analysis comprised measurement of FAF horizontal inner (IR) and outer (OR) hyperautofluorescent ring diameter; SD-OCT ellipsoid zone (EZ) and external limiting membrane (ELM) width, normalised EZ reflectance; AOSLO foveal cone density and intact macular photoreceptor mosaic (IMPM) diameter. Thirty-two eyes from 16 patients (mean age ± SD, 36.0 ± 14.2 years) with *USH2A*-associated Usher syndrome type 2 (*n* = 14) or non-syndromic RP (*n* = 2) met the inclusion criteria. Spatial alignment was observed between IR-EZ and OR-ELM diameters/widths (*p* < 0.001). The IMPM border occurred just lateral to EZ loss (*p* < 0.001), although sparser intact photoreceptor inner segments were detected until ELM disruption. EZ width and IR diameter displayed a biphasic relationship with cone density whereby slow cone loss occurred until retinal degeneration reached ~1350 μm from the fovea, beyond which greater reduction in cone density followed. Normalised EZ reflectance and cone density were significantly associated (*p* < 0.001). As the strongest correlate of cone density (*p* < 0.001) and best-corrected visual acuity (*p* < 0.001), EZ width is the most sensitive biomarker of structural and functional decline in *USH2A* retinopathy, rendering it a promising trial endpoint.

## 1. Introduction

Retinitis pigmentosa (RP) is the encompassing term for a heterogeneous group of progressive inherited retinal diseases (IRDs) characterised by primary degeneration of rod photoreceptors with subsequent cone photoreceptor loss [1]. It manifests symptomatically as nyctalopia followed by concentric visual field constriction, leading to loss of visual acuity upon macular involvement. Funduscopic examination typically reveals mid-peripheral bone-spicule pigmentation, vascular attenuation and waxy optic disc pallor. Approximately 70% of RP cases arise without associated extra-ocular disease (non-syndromic) [1], whilst the remainder occur in conjunction with systemic abnormalities (syndromic) of which Usher syndrome is most prevalent [2].

Usher syndrome (USH) is an autosomal recessive disorder that exhibits varying degrees of RP-related visual loss, sensorineural hearing loss and vestibular dysfunction [3]. It is the most common cause of hereditary deaf–blindness worldwide [4], with an estimated prevalence of 4 to 17 cases per 100,000 people [2,5]. USH is clinically and genetically heterogeneous, and is classified into three subtypes based on age of onset, severity and vestibular features [6]. Of the 16 genes implicated in its pathogenesis [7], disease-causing sequence variants in *USH2A* are the commonest cause of USH although they can also lead to a non-syndromic RP (NSRP) phenotype [8,9]. This 72-exon gene (OMIM #608400) encodes the protein usherin, which is essential for normal retinal photoreceptor, cochlear hair cell and Meissner’s corpuscle function [10,11].

The elucidation of USH-associated genes and their pathogenic variants has enabled precise genotype–phenotype correlation using multimodal retinal imaging [12,13,14]. Together with psychophysical measures of visual function (best-corrected visual acuity (BCVA), colour vision, visual fields) and electroretinography (ERG), this provides insight into structure–function relationships in disease [15,16,17,18,19]. Fundus autofluorescence (FAF) and spectral-domain optical coherence tomography (SD-OCT) are established and widely accessible retinal imaging techniques for the assessment of RP. USH classically displays a hyperautofluorescent ring pattern on FAF prior to foveal involvement [20]. This ring is postulated to circumscribe regions with relatively preserved outer retinal structure and photopic function, as longitudinal reductions in its diameter and area are closely linked to visual field loss and ERG deficits [21,22,23,24,25]. RP causes corresponding concentric loss of the external limiting membrane (ELM) and ellipsoid zone (EZ) layers on OCT [20,22,23,24,25,26,27,28]. These outer retinal hyperreflective bands signify the structural integrity of macular photoreceptors [29], hence decreased band widths correlate with decline in visual function [20,22,23,25,26,28]. Since the shortening of photoreceptor outer segments constitutes the earliest histopathological change in RP [30], OCT assessment for outer retinal disruption is an important diagnostic tool in USH alongside ERG and molecular testing.

Cellular imaging using adaptive optics (AO) has provided insight into photoreceptor morphology in RP [31,32,33], and more specifically in USH [34,35]. Recent adaptive optics scanning laser ophthalmoscopy (AOSLO) devices are designed with multidetector imaging capabilities (confocal [36], dark-field [37], split-detection [38]), and have highlighted a distinct cellular phenotype in USH relative to other IRDs. Sun et al. [35] demonstrated significantly reduced cone density with increased prevalence of non-waveguiding cells in eight USH patients, compared to NSRP and healthy controls. However, decompensatory loss in BCVA to an abnormal level is preceded by a 38–62% decline in foveal cone density from mean normal values [35,39], deeming acuity an insensitive metric of disease advancement. Previous multimodal imaging applying AO in RP has revealed blurred cones of decreased density and increased spacing at the FAF hyperautofluorescent ring [40]. Macular cone loss has corresponded to both an intact [35,41], and disrupted [42,43,44], OCT EZ band, whilst reduction in outer nuclear layer (ONL) thickness may offer greater utility as a marker of structural disturbance [42,45].

There are currently no approved treatments available to prevent USH-associated visual loss. However, with an increasing number of therapeutic trials in progress, and planned [46], better understanding of how photoreceptor morphology relates to more established clinical measures is necessary for selection of sensitive and reliable surrogate endpoints. In this study, we perform the first multimodal retinal imaging correlation of FAF, SD-OCT and AOSLO (confocal and split-detection) in a molecularly confirmed cohort with *USH2A*-RP. We also investigate the relationship of imaging metrics with age and visual function to identify biomarkers of disease progression for future interventional trials.

## 2. Results

### 2.1. Subject Demographics and Genetic Results

Sixteen subjects (mean age ± SD, 36.0 ± 14.2 years; range, 19–55 years) comprised the study cohort following exclusion of those with inadequate image quality (*n* = 8) or an incomplete AOSLO montage (*n* = 1) (Table 1). Snellen decimal BCVA ranged from 1.25 to 0.33 (mean ± SD, 0.77 ± 0.22), with no statistically significant correlation between this and age (*R*^2^ = 0.149, *p* = 0.14). Genetic testing confirmed biallelic pathogenic variants in *USH2A* among all participants, which clinically manifested as Usher syndrome type 2 (USH2) in 14 patients and NSRP in two patients. The most common variant was c.2299delG (p.Glu767Serfs21*), found in five patients, and all were compound heterozygous.

### 2.2. Clinical Imaging Analysis

**Figure 1:**
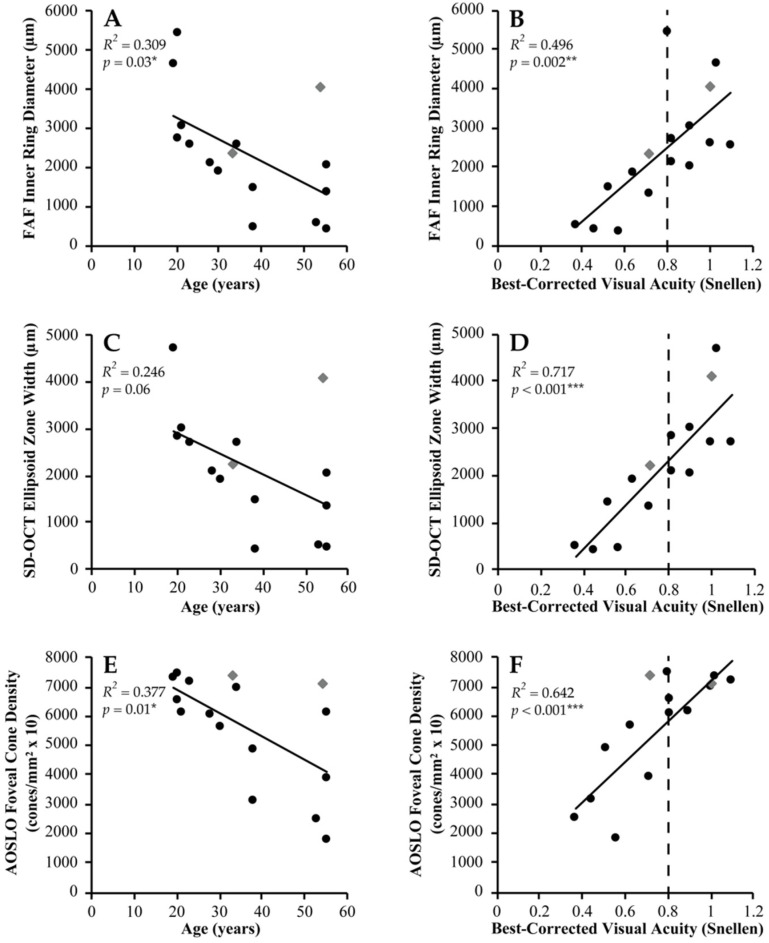
Age and functional correlation of retinal imaging in *USH2A* retinopathy. Scatterplots of retinal imaging metrics in association with (**A**,**C**,**E**) age and (**B**,**D**,**F**) best-corrected visual acuity (BCVA) in *USH2A*-associated Usher syndrome (black circles) and non-syndromic retinitis pigmentosa (grey diamonds) are shown. (**A**,**B**) Fundus autofluorescence (FAF) inner ring diameter (*n* = 16), (**C**,**D**) spectral-domain optical coherence tomography (SD-OCT) ellipsoid zone width (*n* = 15), and (**E**,**F**) adaptive optics scanning laser ophthalmoscopy (AOSLO) foveal cone density (*n* = 16) display the strongest correlations with age and visual function in their respective modalities. BCVA is presented in Snellen decimal format with normal defined as ≥0.80 (6/7.5) (dashed black line). A line of best fit (solid black line) is shown in each graph. *R*^2^ and *p*-values for each correlation are shown, with asterisks denoting the degree of statistical significance.

All subjects exhibited an FAF hyperautofluorescent ring pattern, with mean inner ring (IR; central hypoautofluorescent area) and outer ring (OR; central hypoautofluorescent area plus surrounding hyperautofluorescent ring) diameters ± SD of 2352 ± 1443 μm and 3472 ± 2007 μm, respectively. Intact OCT foveal EZ and ELM bands were detectable in all but extended beyond the scanning window in one and two subjects, respectively, who were excluded from associated analyses. Mean EZ and ELM widths ± SD were 2155 ± 1254 μm and 2874 ± 1055 μm, respectively. Mean normalised EZ reflectance ± SD was 5.79 ± 2.64. All metrics displayed a greater association with BCVA than with age, among which EZ width was strongest (*R*^2^ = 0.717, *p* < 0.001) (Figure 1, Table S1).

### 2.3. Photoreceptor Mosaic Analysis

The qualitative appearance of the AOSLO residual cone mosaic varied considerably, ranging from contiguous to complete loss of outer retinal structure, with numerous hyporeflective confocal areas observed in all. The 64 regions of interest (ROIs) quantitatively analysed (two per eye) consisted of 41 split-detection and 23 confocal images. Mean foveal cone density ± SD at 100 μm eccentricity was 56,230 ± 18,585 cones/mm^2^ (range, 17,700–74,525 cones/mm^2^). Mean intact macular photoreceptor mosaic (IMPM) diameter ± SD was 2754 ± 1840 μm, and was significantly associated with cone density (*R*^2^ = 0.609, *p* < 0.001) (Table S2). Foveal cone density was the strongest correlate of age among all metrics (*R*^2^ = 0.377, *p* = 0.01).

### 2.4. Multimodal Imaging Correlations

#### 2.4.1. FAF versus SD-OCT

**Figure 2:**
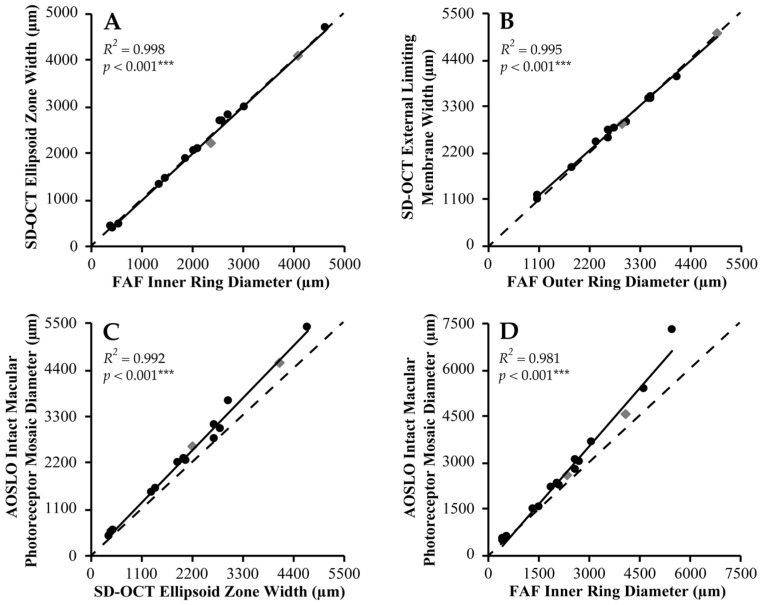
Spatial correlation between retinal imaging in *USH2A* retinopathy. Scatterplots of association between structural parameters on fundus autofluorescence (FAF), spectral-domain optical coherence tomography (SD-OCT) and adaptive optics scanning laser ophthalmoscopy (AOSLO) in *USH2A*-associated Usher syndrome (black circles) and non-syndromic retinitis pigmentosa (grey diamonds) are shown. (**A**,**B**) FAF versus SD-OCT shows close agreement between inner ring diameter and ellipsoid zone width (*n* = 15), and outer ring diameter and external limiting membrane width (*n* = 14). (**C**) SD-OCT versus AOSLO shows linear association between ellipsoid zone width and intact macular photoreceptor mosaic diameter (*n* = 15), similar to that in (**D**) FAF versus AOSLO between inner ring and intact macular photoreceptor mosaic diameters (*n* = 16). Lines of best fit (solid black line) and unity (*x* = *y*, dashed black line) are shown in each graph. *R*^2^ and *p*-values for each correlation are shown, with asterisks denoting the degree of statistical significance.

**Figure 3:**
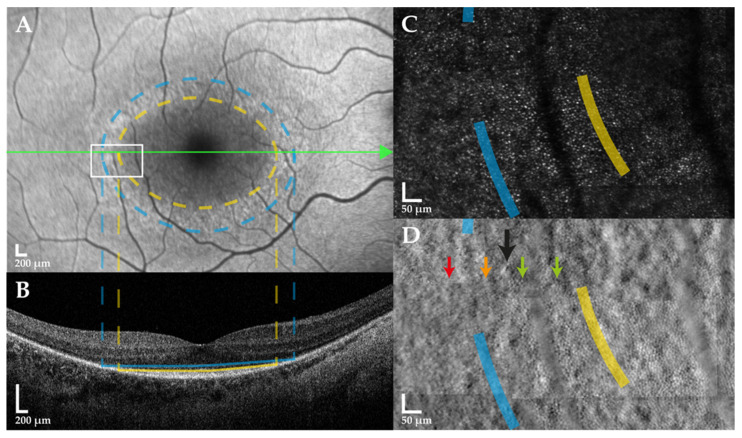
Multimodal retinal imaging in *USH2A* retinopathy. Fundus autofluorescence (FAF), spectral-domain optical coherence tomography (SD-OCT) and adaptive optics scanning laser ophthalmoscopy (AOSLO) images of the right eye in a 34-year-old female with *USH2A*-associated Usher syndrome type 2 are shown. The lateral borders of the inner (dashed yellow circle) and outer (dashed blue circle) hyperautofluorescent ring in (**A**) FAF spatially align with the lateral extents of the ellipsoid zone (solid yellow line) and external limiting membrane (solid blue line) bands, respectively, in the (**B**) SD-OCT horizontal B-scan through the foveal centre (horizontal green arrow). A magnified view of the (**C**) confocal and (**D**) split-detection AOSLO montage at the hyperautofluorescent ring (white rectangle) shows regular cone inner segments (vertical green arrows) until generalised disruption of the photoreceptor mosaic (vertical black arrow), beyond which cells are sparser (vertical amber arrow) followed by complete loss outside the outer ring (vertical red arrow).

Linear associations were observed between IR diameter and EZ width (*R*^2^ = 0.998, *p* < 0.001), and OR diameter and ELM width (*R*^2^ = 0.995, *p* < 0.001) (Table S3), whereby both parameters were approximately equivalent (Figure 2A,B). Mean difference ± SD between IR diameter and EZ width was −8 ± 66 μm, and between OR diameter and ELM width was −39 ± 76 μm. Spatial correspondence between FAF ring borders and OCT band disruption is supported by multimodal image alignment (Figure 3A,B).

#### 2.4.2. SD-OCT versus AOSLO

**Figure 4:**
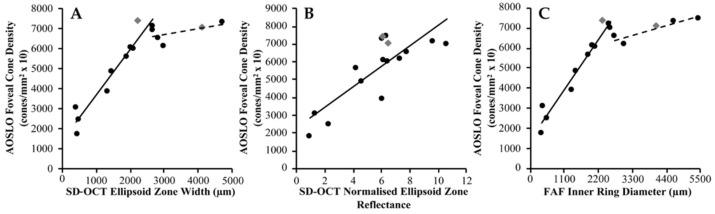
Clinical retinal imaging correlation with photoreceptor density in *USH2A* retinopathy. Scatterplots of metrics from (**A**,**B**) spectral-domain optical coherence tomography (SD-OCT) and (**C**) fundus autofluorescence (FAF) imaging in association with foveal cone density in *USH2A*-associated Usher syndrome (black circles) and non-syndromic retinitis pigmentosa (grey diamonds) are shown. (**A**) SD-OCT ellipsoid zone width (*n* = 15) and (**C**) FAF inner ring diameter (*n* = 16) demonstrate a biphasic relationship with foveal cone density, whereby reduction to ~2700 μm is associated with slow cone loss (dashed black line) below which faster decline in cone density occurs (solid black line). (**B**) SD-OCT normalised ellipsoid zone reflectance (*n* = 16) displays linear correlation with foveal cone density, with a line of best fit shown (solid black line).

EZ width and IMPM diameter showed linear association (*R*^2^ = 0.992, *p* < 0.001) with a mean difference ± SD of −292 ± 201 μm (EZ loss closer to the fovea than gross mosaic disruption) (Figure 2C). Cone inner segments were observed beyond the IMPM border, albeit sparser, but terminated at the point of ELM disruption. EZ width displayed a biphasic relationship with foveal cone density, whereby its reduction to ~2700 μm was associated with slow cone loss below which greater decline in cone density occurred (Figure 4A). Normalised EZ reflectance and cone density were significantly correlated (*R*^2^ = 0.658, *p* < 0.001) (Figure 4B). EZ width was the strongest correlate of foveal cone density among all metrics (*R*^2^ = 0.738, *p* < 0.001).

#### 2.4.3. FAF versus AOSLO

IR and IMPM diameters were linearly associated (*R*^2^ = 0.981, *p* < 0.001) with a mean difference ± SD of −402 ± 457 μm (IR border closer to the fovea than gross mosaic disruption) (Figure 2D). AOSLO mosaic superimposition on FAF revealed loss of contiguous cone inner segments at the hyperautofluorescent ring, with complete absence beyond the OR border (Figure 3C,D). IR diameter and foveal cone density exhibited a similar biphasic relationship as EZ width and the latter, since IR constriction to less than ~2700 μm diameter caused increased cone loss (Figure 4C).

## 3. Discussion

This phenotypic analysis constitutes the largest AO-aided retinal assessment of Usher syndrome to date. In our cohort, we report spatial alignment between multimodal imaging metrics, a biphasic relationship between EZ width/IR diameter and foveal cone density, and correlation between EZ reflectance and cone density. As the strongest correlate of foveal cone density and BCVA, EZ width emerged as the most sensitive biomarker of cone loss and functional decline.

Conventional retinal imaging evaluation revealed a hyperautofluorescent ring in all participants, which is the earliest of three abnormal FAF patterns signifying disease stage in USH [20,47]. The ring demarcates centrally intact retina from peripheral degeneration by highlighting retinal pigment epithelium (RPE) lipofuscin accumulation in metabolically active tissue [48]. Structural alignment between FAF and OCT indicates photoreceptor inner segment preservation with outer segment loss at the hyperautofluorescent ring, due to corresponding intact ELM and absent EZ bands. The ELM represents junctional complexes between photoreceptor inner segments and Müller cells, whilst contention exists whether the EZ originates from inner segment ellipsoids or inner segment–outer segment junctions [29]. Increased autofluorescence in RP may thus partially arise from abnormal bisretinoid levels within the photoreceptor itself, which could contribute to degeneration [20].

The arrival of AO-based cellular in vivo retinal imaging has enabled detailed genotype–phenotype characterisation [49], longitudinal disease monitoring [33,50], and treatment response quantification in IRDs using photoreceptor-based metrics [33,51]. Foveal cone density, a widely applied mosaic metric, was reduced in all participants compared to normative values at 100–150 μm retinal eccentricity derived from histological work and AO mapping studies [52,53]. This occurred despite appearances of no foveal involvement by retinal degeneration on clinical imaging. The confocal photoreceptor mosaic also contained numerous non-waveguiding cone outer segments, previously noted to arise more than seen in healthy subjects [35]. Whilst dysflective cones may constitute the earliest phase of RP-related degeneration [32,43], their occurrence in normal eyes with normal perceptual sensitivity raises ambiguity regarding their significance [54]. Nevertheless, complementary use of confocal and split-detection AOSLO is advocated for accurate assessment of the remnant mosaic, as the latter visualises photoreceptor inner segments irrespective of outer segment integrity [38]. This is important in the context of an interventional trial, where distinguishing between a disrupted or altogether absent cell may determine treatment success. Comparison between photoreceptor mosaics showed larger-sized remnant inner segments in participants with lower cone densities than those with higher densities, which may be secondary to the degenerative process itself or loss of neighbouring cones [51]. However, neither photoreceptor reflectivity nor size were formally quantified in this study and warrant future analysis.

Correlation of photoreceptor topography with OCT highlighted interesting relationships that may advance the current understanding of USH pathophysiology. In accordance with existing literature [20,22,23,24,25,26,27,28], most subjects exhibited a degree of concentric EZ and ELM loss signifying encroachment of retinal degeneration into the OCT scanning window. The associated biphasic decline in foveal cone density was dependent on the distance between the degenerating retinal edge and fovea, which caused more rapid cone loss when less than ~1350 μm radially. This may be attributed to the exponential increase in normal cone packing density from this eccentricity (~4.5°) to the fovea [52,55]. A lower rod to cone ratio may assist in propagation of the apoptotic signal to foveal cones within “intact” retina via microglial activation [56], or lead to cone death secondary to loss of the rod-derived cone viability factor [57]. In addition, the association between EZ reflectance and foveal cone density is similar to findings in other maculopathies [58,59]. EZ reflectance is believed to predominantly arise from mitochondrial packing in the photoreceptor inner segment ellipsoid [29]. Its correlation with cone density, largely derived from inner segment identification in this study, is, therefore, rationalised. However, other groups employed AO flood illumination ophthalmoscopy for cone density analysis, which, like confocal AOSLO, only visualises waveguiding photoreceptor outer segments [58,59]. This suggests both inner segment integrity and outer segment reflectivity are contributory to EZ reflectance.

Our multimodal image alignment findings affirm previous USH cellular characterisation of generalised photoreceptor mosaic disruption adjacent to EZ loss, and inner segment termination at ELM collapse [34,35]. However, SD-OCT is limited to detecting disease within the last 30 degrees of the posterior pole, whereas FAF affords a wider field of view for monitoring less advanced RP. FAF-AOSLO image superimposition implicates the hyperautofluorescent ring as a transition zone at which significant mosaic disturbance occurs, and beyond which no intact inner segments exist. Both ELM width and OR diameter are thus beneficial as biomarkers for estimating the retinal area with potential for post-interventional cone recovery, particularly the latter in early disease.

Evaluation of treatment efficacy requires sensitive and reliable trial endpoints to detect slowing or halting of disease progression. Measures of visual function are advocated as surrogate endpoints for clinical trials targeting ocular disease by the US Food and Drug Administration (FDA), since they directly impact patients’ quality of life [60]. However, the occurrence of microstructural retinal abnormalities without change in vision renders functional endpoints insensitive [33,35,39], as we observed a reduction in mean foveal cone density of ~50% from normal at 100 μm eccentricity before resulting in abnormal BCVA (6/7.5 or less) [52,53]. Moreover, observational studies of RP indicate that reliably measurable change in visual function is only possible after several years because photoreceptor loss occurs so gradually [25,61,62]. Although AOSLO-based metrics are the most sensitive, their uptake is hindered by limited availability, lack of multi-centre standardisation, and time-consuming image analysis [63]. Our study thus establishes EZ width as the most promising trial endpoint for *USH2A*-associated RP based upon its association with foveal cone density and BCVA. In keeping with this, EZ width demonstrated good functional correlation and repeatability in previous RP studies [64,65,66], and was recommended at the National Eye Institute–FDA endpoints workshop on IRDs [60].

Our study has several limitations. Despite the improved lateral resolution AOSLO possesses compared to SD-OCT, individual photoreceptors at the foveal centre were unable to be resolved. Foveal cone density was, therefore, calculated by sampling targeted retinal areas but would be better represented by density deviation mapping across foveal strips. Consequently, our findings cannot be extrapolated to other retinal locations. Variations in cone density related to age, retinal meridian and sampling window size may also occur [55,67], however were not addressed in this study. Due to our small sample size and cross-sectional study design, differences between the cellular phenotype of *USH2A*-associated NSRP and USH2 could not be assessed, although the former is believed to induce a milder disease course [9,13,14,15,16,17,18,19].

## 4. Materials and Methods

### 4.1. Study Population

Twenty-five patients with a genetic and clinical diagnosis of *USH2A*-associated USH2 (*n* = 21) or NSRP (*n* = 4) were recruited from Moorfields Eye Hospital between May 2017 and December 2018 in this prospective, non-interventional image analysis study. The study adhered to the tenets of the Declaration of Helsinki, received approval from a National Research Ethics Service (NRES) ethics committee, and was performed with written informed consent from all participants. Both eyes from each subject were analysed. Exclusion criteria included presence of alternative causes for visual loss, concurrent retinal disease, and insufficient image quality.

All patients underwent molecular genetics testing, comprehensive ophthalmological examination (including slit-lamp biomicroscopy), and multimodal retinal imaging (FAF, SD-OCT, confocal and split-detection AOSLO). LogMAR BCVA was assessed with an Early Treatment of Diabetic Retinopathy Study chart and converted into Snellen decimal units to facilitate comparison. Axial length measurements were obtained using the Zeiss IOL Master (Carl Zeiss Meditec, Dublin, CA, USA) to determine the micrometres per degree scale for each eye.

### 4.2. Fundus Autofluorescence and Spectral-Domain Optical Coherence Tomography

Macular 30° × 30° BluePeak FAF and 20° × 20° volumetric SD-OCT scans were simultaneously acquired from each eye using the Spectralis HRA+OCT (Heidelberg Engineering GmbH, Heidelberg, Germany). Automatic real-time tracking was activated for noise reduction, whereby alignment of up to 100 frames produced each final image. SD-OCT volumes consisted of 49, 97 or 193 horizontal B-scans, from which the central foveal frame was selected as that with the least residual inner retinal tissue and thickest ONL.

**Figure 5:**
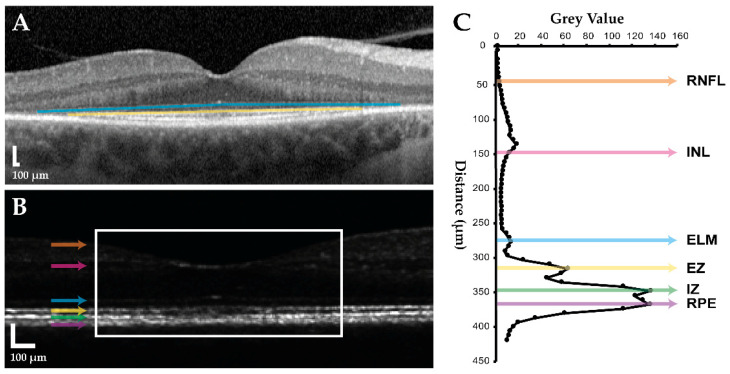
Spectral-domain optical coherence tomography (SD-OCT) image analysis. SD-OCT imaging of the right eye in a 54-year-old female with *USH2A*-associated non-syndromic retinitis pigmentosa is shown. A horizontal B-scan through the foveal centre is used in (**A**) logarithmic form for outer retinal band width measurement, and (**B**) linear form for longitudinal reflectivity profile (LRP) analysis. External limiting membrane (ELM, solid blue line) and ellipsoid zone (EZ, solid yellow line) widths are measured before B-scan linear transformation, after which an LRP is generated from a 1000 μm-wide window centred at the foveal pit (white rectangle). Coloured arrows indicate the retinal layers corresponding to peaks in the resulting (**C**) LRP trace: orange, retinal nerve fibre layer (RNFL); pink, inner nuclear layer (INL); blue, ELM; yellow, EZ; green, interdigitation zone (IZ); purple, retinal pigment epithelium (RPE).

Heidelberg Eye Explorer software (Heidelberg Engineering GmbH) was employed for image analysis using its integrated micrometre caliper. IR and OR horizontal diameters were measured on FAF, whilst intact EZ and ELM widths were measured on the central SD-OCT B-scan (Figure 5A). All measurements were taken twice by the same grader to ensure accuracy and adjudicated by a second grader.

Longitudinal reflectivity profile (LRP) analysis was also performed on the central B-scan to assess normalised EZ reflectance using ImageJ (National Institutes of Health, Bethesda, MD, USA) [68,69]. The selected frame was transformed from logarithmic to linear scale, and an LRP generated from a 1000 μm-wide window centred at the foveal pit (Figure 5B). Six layers were manually identified in the resulting trace: retinal nerve fibre layer (RNFL), inner nuclear layer (INL), ELM, EZ, interdigitation zone (IZ), and RPE (Figure 5C). Normalised EZ reflectance was calculated by dividing mean EZ reflectance by mean inner retinal reflectance (RNFL to INL).

### 4.3. Adaptive Optics Scanning Laser Ophthalmoscopy

**Figure 6:**
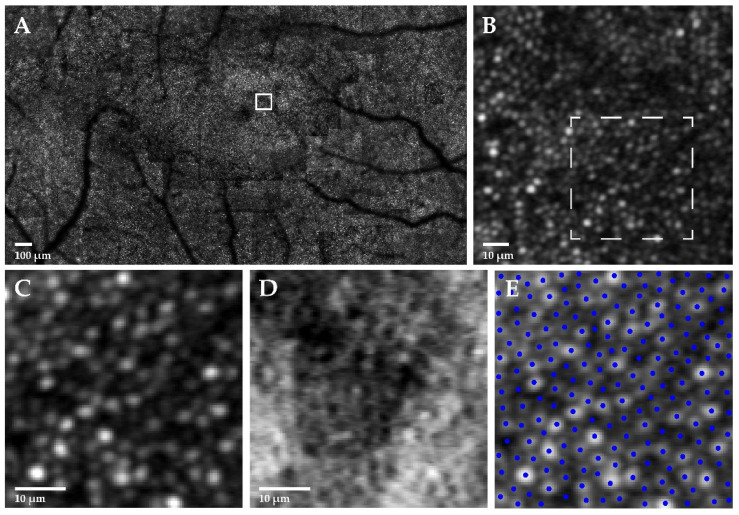
Adaptive optics scanning laser ophthalmoscopy (AOSLO) cone density analysis. AOSLO imaging of the right eye in a 33-year-old female with *USH2A*-associated non-syndromic retinitis pigmentosa is shown. (**A**) 1.5° field of view confocal photoreceptor mosaic with a 100 × 100 μm cone sampling window (solid white square) applied at 100 μm eccentricity to generate (**B**) a region of interest (ROI). A magnified section of the ROI (dashed white square) is displayed in (**C**) confocal and (**D**) split-detection modalities, with (**E**) manual cone identification (blue circles) performed in the former.

All subjects underwent simultaneous confocal and split-detection retinal imaging with a custom-built AOSLO system [37,38,70]. Image sequences were acquired using a 790 nm superluminescent diode subtended at 1° foveal or 1.5° parafoveal square field of view (FOV) with perfect spatial register between detection modes. These were processed to remove distortion due to the sinusoidal motion of the resonant scanner by estimating distortion of a calibrated Ronchi ruling before resampling the images over a grid of equally spaced pixels. Each sequence was registered using a reference frame and strip motion estimation to generate a final image with improved signal-to-noise ratio [71]. Registered images were assembled into a montage of the photoreceptor mosaic in a semi-automated manner using MATLAB (The MathWorks Incorporated, Natick, MA, USA) and Adobe Photoshop (Adobe Systems Incorporated, San Jose, CA, USA) [72]. The relative locus of fixation was defined as the centre of the image obtained when the patient was instructed to look at the centre of the scanning raster. AOSLO image analysis comprised measurement of foveal cone density (Figure 6), and IMPM diameter.

To assess cone density, two 100 × 100 μm cone sampling windows were applied at 100 μm eccentricity in each montage after scaling according to axial length. Confocal and split-detection images from each ROI were individually compared to determine the modality with better image quality and more detectable cones. Selected images were presented to the grader in a random order by MATLAB for manual cone identification, and reviewed by a second grader for misidentified or missed cones. A mean value for foveal cone density was calculated from two ROIs per eye. To assess IMPM diameter, the 1.5° FOV split-detection montage of each eye was examined for lateral points of structural disruption along a horizontal axis passing through the fovea, and the distance between these points was measured.

### 4.4. Statistical Analysis

Statistical analysis was conducted using Microsoft Excel version 16.51 (Microsoft Corporation, Redmond, WA, USA). Correlation with age and BCVA, or between metrics, was assessed using the coefficient of determination (*R*^2^). A mean value combining paired eyes was calculated for each metric as eyes from the same subject were not considered as independent samples. A *p*-value of <0.05 was considered statistically significant.

## 5. Conclusions

Interventional trials directed towards RP in USH are underway, with more in the pipeline. The development of robust retinal imaging protocols and analysis tools is essential for identifying therapeutic potential, stratifying eligible candidates, and assessing treatment response. To facilitate this, a clear understanding of how imaging markers relate to retinal microstructure and visual function in disease is necessary. Upon direct correlation of established retinal imaging modalities, FAF and SD-OCT, with the photoreceptor anatomy underlying RP, EZ width is most closely associated with structural and functional status among all metrics. Until AOSLO overcomes the challenge of transitioning from research laboratory to widespread clinical use, EZ width is the most suitable trial surrogate endpoint in *USH2A* retinopathy. Regardless, this is an exciting time for the emergence of RP-targeted therapies, assisted by rapid advances in retinal imaging capabilities.

## Figures and Tables

**Table 1:** Summary of subject demographics, genetic results and clinical characteristics. Genetic and clinical data of the 16 patients forming the study cohort with biallelic pathogenic variants in *USH2A* are shown. Axial length measurements are provided in millimetres. Best-corrected visual acuity (BCVA) is shown in Snellen decimal format with normal defined as ≥0.80 (6/7.5). F, female; M, male; NSRP, non-syndromic retinitis pigmentosa; OD, right eye; OS, left eye; USH2, Usher syndrome type 2.

Subject	Gender	Age	Phenotype	Variant 1 cDNA	Variant 2 cDNA	Axial Length		BCVA	
				Variant 1 Protein	Variant 2 Protein	OD	OS	OD	OS
S19	F	33	NSRP	c.11864G>A	c.13335_13347delinsCTTG	23.88	23.84	0.80	0.63
				p.Trp3955*	p.Glu4445_Ser4449delinsAspLeu				
S21	M	55	USH2	c.100C>T	c.926C>T	24.07	23.84	0.50	0.63
				p.Arg34*	p.Pro309Leu				
S23	M	55	USH2	c.8834G>A	c.2299delG	24.24	24.25	0.80	0.63
				p.Trp2945*	p.Glu767Serfs21*				
S24	F	55	USH2	c.4821G>A	c.1859G>T	22.27	22.16	0.80	1.00
				p.Trp1607*	p.Cys620Phe				
S31	M	53	USH2	c.4474G>T	c.11047+1G>A	23.32	23.26	0.33	0.40
				p.Glu1492*	Deep intronic variant				
S33	F	38	USH2	c.6862G>T	c.7595–2144A>G	25.36	24.98	0.40	0.63
				p.Glu2288*	Deep intronic variant				
S39	M	30	USH2	c.187C>T	c.4645C>T	22.12	22.20	0.63	0.63
				p.Arg63*	p.Arg1549*				
S40	F	54	NSRP	c.8981G>A	c.13274C>T	23.37	23.21	1.00	1.00
				p.Trp2994*	p.Thr4425Met				
S43	M	38	USH2	c.1876C>T	c.2299delG	23.04	22.97	0.40	0.50
				p.Arg626*	p.Glu767Serfs21*				
S47 ^a^	M	19	USH2	c.163C>T	c.2299delG	26.10	25.44	1.25	0.80
				p.Gln55*	p.Glu767Serfs21*				
S48	F	20	USH2	c.3518C>A	c.920_923dupGCCA	22.12	22.10	0.80	0.80
				p.Ser1173*	p.His308Glnfs16*				
S49 ^a^	M	21	USH2	c.163C>T	c.2299delG	27.09	26.77	0.80	1.00
				p.Gln55*	p.Glu767Serfs21*				
S50 ^b^	M	28	USH2	c.12954C>A	c.10488_10490del	22.35	22.04	1.00	0.63
				p.Tyr4318*	p.Glu3496del				
S51 ^b^	F	34	USH2	c.12954C>A	c.10488_10490del	24.47	24.76	1.20	0.80
				p.Tyr4318*	p.Glu3496del				
S52	M	23	USH2	c.11065C>T	c.7645_7661del	23.62	23.55	1.20	1.00
				p.Arg3689*	p.Met2549Alafs3*				
S55	F	20	USH2	c.7814C>G	c.2299delG	23.72	23.80	0.63	1.00
				p.Ser2605*	p.Glu767Serfs21*				

^a, b^ Siblings.

## Data Availability

The data presented in this study are available on request from the corresponding author.

## Supplementary Materials

The following supporting information can be downloaded at: https://www.mdpi.com/article/10.3390/ijms23084198/s1.
