# Supplementary material for: Investigating Biomarkers for USH2A Retinopathy Using Multimodal Retinal Imaging
Source: Int J Mol Sci. 2022 Apr 11;23(8):4198. doi: 10.3390/ijms23084198 (PMC9024786; doi:10.3390/ijms23084198)
Supplement: Supplementary file 1 [file ijms-23-04198-s001.zip › ijms-1639834-supplementary.pdf]

International Journal of *Molecular Sciences*

MDPI

Article

Investigating Biomarkers for *USH2A* Retinopathy Using Multimodal Retinal Imaging

Jasdeep S. Gill [1], Vasileios Theofylaktopoulos [1], Andreas Mitsios [1,2], Sarah Houston [1], Ahmed M. Hagag [1,2], Adam M. Dubis [1,2,3,†] and Mariya Moosajee [1,2,4,*,†]

1 Institute of Ophthalmology, University College London, London EC1V 9EL, UK; j.gill.17@ucl.ac.uk (J.S.G.); v.theofylaktopoulos@ucl.ac.uk (V.T.); andreas.mitsios@ucl.ac.uk (A.M.); sarah.houston.16@ucl.ac.uk (S.H.); a.hagag@ucl.ac.uk (A.M.H.); a.dubis@ucl.ac.uk (A.M.D.)
2 NIHR Moorfields Biomedical Research Centre, Moorfields Eye Hospital NHS Foundation Trust, London EC1V 2PD, UK
3 Global Business School for Health, University College London, London WC1E 6BT, UK
4 Great Ormond Street Hospital for Children NHS Foundation Trust, London WC1N 3JH, UK
* Correspondence: m.moosajee@ucl.ac.uk; Tel.: +44-207-608-6971
† These authors contributed equally to this work.

Citation: Gill, J.S.; Theofylaktopoulos, V.; Mitsios, A.; Houston, S.; Hagag, A.M.; Dubis, A.M.; Moosajee, M. Investigating Biomarkers for *USH2A* Retinopathy Using Multimodal Retinal Imaging. *Int. J. Mol. Sci.* **2022**, *23*, 4198. https://doi.org/10.3390/ijms23084198

Academic Editor: Stephanie C. Joachim

Received: 1 March 2022
Accepted: 6 April 2022
Published: 11 April 2022

Publisher's Note: MDPI stays neutral with regard to jurisdictional claims in published maps and institutional affiliations.

Copyright: © 2022 by the authors. Submitted for possible open access publication under the terms and conditions of the Creative Commons Attribution (CC BY) license (https://creativecommons.org/licenses/by/4.0/).

Tables S1-S3. Correlation with age, best-corrected visual acuity (Snellen), and between metrics is shown using the coefficient of determination (R^2). The number of subjects included in each analysis is denoted by *n*, with a mean value combining paired eyes calculated for each metric. $p < 0.05$ is considered statistically significant.

Table S1. Age and functional correlation of retinal imaging in *USH2A* retinopathy.

Imaging Modality	Correlation of:	*n*	with Age		with BCVA	
			R^2	*p*	R^2	*p*
FAF	IR Diameter	16	0.309	0.03*	0.496	0.002**
	OR Diameter	16	0.288	0.03*	0.353	0.02*
SD-OCT	EZ Width	15	0.246	0.06	0.717	< 0.001***
	ELM Width	14	0.090	0.30	0.653	< 0.001***
	EZ Reflectance	16	0.220	0.07	0.712	< 0.001***
AOSLO	Foveal Cone Density	16	0.377	0.01*	0.642	< 0.001***
	IMPM Diameter	16	0.300	0.03*	0.409	0.008**

Table S2. Intramodal retinal imaging correlation in *USH2A* retinopathy.

Imaging Modality	Correlation of:	*n*	R^2	*p*
FAF	IR Diameter with OR Diameter	16	0.928	< 0.001***
SD-OCT	EZ Width with ELM Width	14	0.962	< 0.001***
	EZ Width with EZ Reflectance	15	0.451	0.006**
	ELM Width with EZ Reflectance	14	0.592	0.001**
AOSLO	Foveal Cone Density with IMPM Diameter	16	0.609	< 0.001***

Table S3. Multimodal retinal imaging correlation in *USH2A* retinopathy.

Imaging Modality	Correlation of:	*n*	R^2	*p*
FAF versus SD-OCT	IR Diameter with EZ Width	15	0.998	< 0.001***
	IR Diameter with ELM Width	14	0.963	< 0.001***
	IR Diameter with EZ Reflectance	16	0.331	0.02*
	OR Diameter with EZ Width	15	0.896	< 0.001***
	OR Diameter with ELM Width	14	0.995	< 0.001***
	OR Diameter with EZ Reflectance	16	0.196	0.09
SD-OCT versus AOSLO	EZ Width with Foveal Cone Density	15	0.738	< 0.001***
	EZ Width with IMPM Diameter	15	0.992	< 0.001***
	ELM Width with Foveal Cone Density	14	0.693	< 0.001***
	ELM Width with IMPM Diameter	14	0.963	< 0.001***
	EZ Reflectance with Foveal Cone Density	16	0.658	< 0.001***
	EZ Reflectance with IMPM Diameter	16	0.271	0.04*
FAF versus AOSLO	IR Diameter with Foveal Cone Density	16	0.695	< 0.001***
	IR Diameter with IMPM Diameter	16	0.981	< 0.001***
	OR Diameter with Foveal Cone Density	16	0.506	0.002**
	OR Diameter with IMPM Diameter	16	0.941	< 0.001***

Abbreviations: AOSLO, adaptive optics scanning laser ophthalmoscopy; BCVA, best-corrected visual acuity; ELM, external limiting membrane; EZ, ellipsoid zone; FAF, fundus autofluorescence; IMPM, intact macular photoreceptor mosaic; IR, inner hyperautofluorescent ring; OR, outer hyperautofluorescent ring; SD-OCT, spectral-domain optical coherence tomography.
